# Anatomical study of peroneal longus and brevis innervation and its spatial relationship with trigger points

**DOI:** 10.1590/acb412626

**Published:** 2026-06-29

**Authors:** Nazareth Cristina Delantonia, Lucas Yongsoo Park, Flávio Hojaij, Mauro Andrade, Alfredo Luiz Jacomo, Flavia Emi Akamatsu Jacomo

**Affiliations:** 1 Universidade de São Paulo – Faculty of Medicine – Department of Surgery – São Paulo (SP) – Brazil.

**Keywords:** Trigger Points, Muscle, Anatomy, Pain

## Abstract

**Purpose:**

Myofascial pain syndrome (MPS) is a common cause of chronic musculoskeletal pain. Long fibular syndrome is a symptom of lower-limb MPS. Research has associated MPS with motor end plates in the innervation zone. This study aimed to identify the anatomical entry points of the superficial peroneal nerve (SPN) in the peroneal longus (PL) and peroneal brevis (PB) muscles through dissection.

**Methods:**

The PL and PB muscles of 12 cadavers were dissected to identify the nerve entry points, which were mapped and organized into quadrants. Poisson marginal distribution, identity linkage function, and Bonferroni multiple comparisons were used for statistical analysis. Statistical analysis included summary measures, Student’s and Mann–Whitney group comparisons, Pearson and Spearman correlations. Statistical significance was set at *p* < 0.05.

**Results:**

Quadrant I, followed by quadrant II (upper half of the PL and PB muscles), had the highest number of SPN entry points. Quadrants III and IV (lower half of the PL and PB muscles) had the lowest numbers of nerve entry points and did not differ statistically from each other.

**Conclusion:**

The SPN entrance positions in the PL and PB muscles matched literature trigger points. The anatomical relationship between trigger points can aid healthcare providers in procedures affecting muscle innervation and in pinpointing pain locations through spatial localization.

## Introduction

Public health is significantly affected by musculoskeletal disorders, particularly chronic muscle pain, which affects millions of individuals worldwide^1^. The prevalence of myofascial pain can be challenging to ascertain owing to the absence of definitive diagnostic criteria that must be met by patients. The reported prevalence of myofascial pain is subject to considerable variation, with authors proposing a broad spectrum of estimates. Between 20 and 95% of patients presenting to general medicine physicians and pain management centers with musculoskeletal pain are diagnosed with myofascial pain^2^.

Myofascial trigger points (MTPs) are a defining feature of myofascial pain syndrome (MPS)^3,4^. These points exhibit a sensory component mediated by nociceptors and localized muscle spasms, resulting in pain and limited movement^5^. Excessive release of acetylcholine in the motor endplates has been proposed as the primary factor involved in the development of MTPs. Notably, increased concentrations of acetylcholine in the synaptic cleft, changes in the acetylcholine receptor, and changes in acetylcholinesterase activity are known mechanisms of endplate dysfunction^6-8^. These mechanisms may explain the increased endplate electrical activity observed in active MTPs^9^. One of the most common presentations of MPS of the lower limbs is longus fibular syndrome^10^.

Pathology of the peroneal tendon occurs due to a wide variety of etiologies, including overuse and ankle sprains^11,12^. Peroneal tendon disorders can cause hindfoot and lateral foot pain. There are three primary tendon disorders—peroneal tendonitis, peroneal subluxation, and peroneal tendon tears—, which cause lateral ankle pain and may lead to ankle instability^10^. Forced inversion injuries during ankle injuries without prior pain or dysfunction of the fibular muscle can lead to peroneal rupture, micro-laceration, and scar tissue formation. Peroneal tendon pathology can also occur before injury since the role of the peroneal tendons as active stabilizers of the ankle joint^13^. Excessive use of the peroneal muscles in certain running styles (forefoot or midfoot strike) can cause tendon pathology, leading to ankle injuries^14^. Due to alterations of the ankle position in its frontal plane, locomotion over irregular surfaces (slopes, snow, sand, grass, gravel, etc.) leads to changes in the activation of the tibial and peroneal muscles^15-18^. In the United States of America, approximately two million acute ankle sprains occur annually^12^.

The peroneal longus (PL) muscle is one of the two muscles in the lateral compartment of the lower limb, along with the peroneal brevis (PB) muscle. The lateral compartment receives innervation from the superficial peroneal nerve (SPN; L5–S2)

and is supplied by the anterior tibial and peroneal arteries. This is important for the plantar flexion and eversion of the foot and ankle. The PL muscle is susceptible to several pathologies, including tendonitis, tendon dislocation, subluxation, rupture, and acute and chronic compartment syndromes, which cause lateral ankle pain and may lead to ankle instability^19^.

The peroneal tendons are in the lateral compartment of the leg and include the PL and PB muscles. Both tendons receive innervation from the SPN and perfusion from the peroneal artery. The PB originates on the lateral aspect of the distal fibula and intermuscular septum and inserts into the base of the fifth metatarsal. The PL originates at the proximal fibula and lateral tibia and inserts at the base of the first metatarsal and the medial cuneiform. The tendons occupy a common synovial sheath that runs posterior to the distal fibula; once past the fibula, each tendon has its own synovial sheath. They run in a tunnel bordered by the superior peroneal retinaculum, the posterior fibula with a retromalleolar groove, and the calcaneofibular ligament^10^.

**Figure 1:**
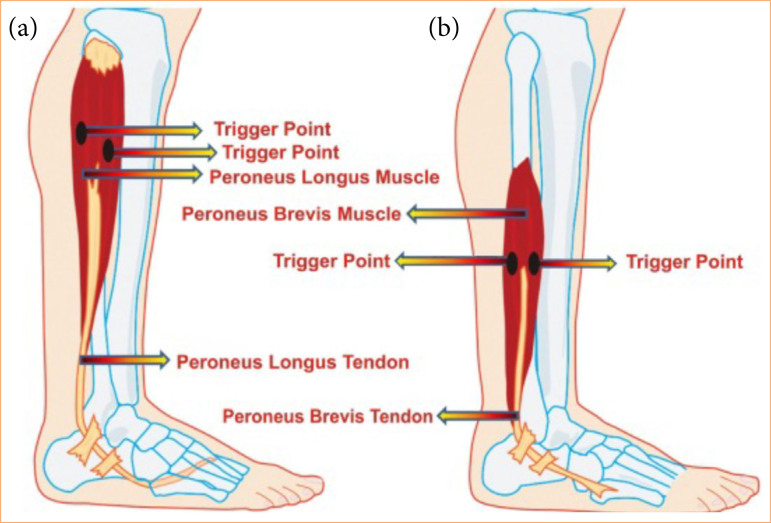
Usual location of trigger points in the (a) peroneal longus and (b) peroneal brevis muscles, right lateral view.

Trigger points (TPs) in the long peroneal muscle are located near the muscle origin, 2–4 cm distal to the head of the fibula, and in the lower third of the muscle belly (Fig. 1). In the PB muscle, the TPs are found in the depth of the longus peroneal tendon, more distally near the junction of the middle and lower thirds of the leg in the upper half of the muscle^5^ (Fig. 1). The referred pain and sensitivity caused by TPs in the PL and PB muscles are concentrated over the lateral malleolus, above, behind, and below it, and extend a short distance along the lateral sides of the foot and the medial third of the leg^5^.

Recently, ultrasonography^20,21^ and magnetic resonance imaging (MRI)^21^ have revealed the presence of MTPs and taut bands, which are localized regions of increased muscular stiffness caused by taut bands, inflammation, or autonomic alterations. Hypoxia and ischemia promote the production of molecules that activate peripheral nociceptors, resulting in pain. This lends credence to Simons’ theory that excessive acetylcholine release causes MTPs by abnormal contractions in the taut band^5,7,20,21^.

Various therapeutic strategies, including invasive and non-invasive techniques such as dry needling, acupuncture, shockwave application, and local infiltration with anesthetics, have been employed in the management of pain related to MTPs. The results vary in the literature, requiring studies to standardize and confirm protocols^21,22^. No studies have correlated innervation of the SPN in the longus and brevis peroneal muscles with MTPs.

This study describes the entry points of the SPN into the PL and PB muscles and their relationship with MTPs, establishing an anatomical guide for the treatment of MPS in the referred muscles and providing a better understanding of conservative and interventional approaches that help standardize the protocols.

## Methods

### Ethical aspects

This study was approved by the Ethics Committee of the Medical School for the Analysis of Research Projects, Protocol No. 5.713.360.

### Anatomical technique

A similar methodology was used by Akamatsu et al.^23^ to evaluate the location of the points of penetration of the nerve branches in the muscle belly via anatomical dissection^23^.

**Figure 2:**
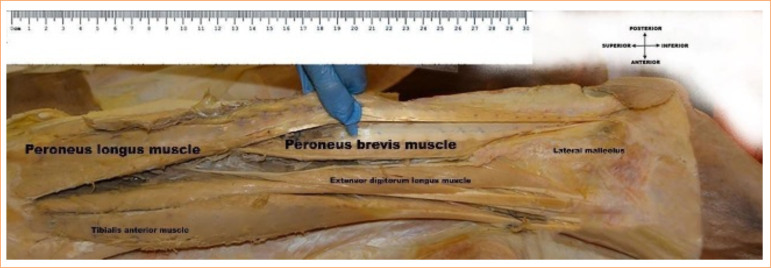
Cadaver in the prone position, left lower limb.

Source: Elaborated by the author Flavia Emi Akamatsu Jacomo.

**Figure 3:**
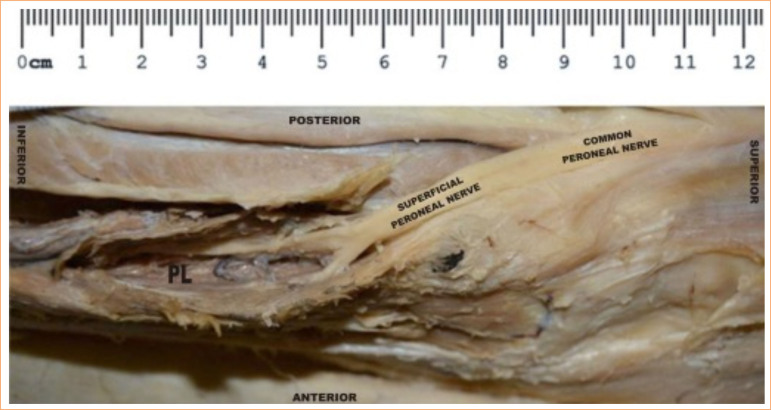
Right lower limb. Superficial peroneal nerve entering the peroneal longus (PL) muscle and giving off its branches at its entrance into the PL muscle.

Source: Elaborated by the author Flavia Emi Akamatsu Jacomo.

Twenty-four PL and PB muscles from cadavers (four male and eight female) donated to the discipline of Human Structural Topography of the Department of Surgery of the university’s medical school were dissected to expose the branches of the SPN and their points of entry into the PL and PB muscles. The cadavers were fixed with a 4% phenolic acid and 0.5% formaldehyde solution. None of the specimens had any deformities or previous manipulation of the leg area. The specimens were positioned in the ventral decubitus position for better visualization of the nerve pathway, and dissection was performed starting with an incision at the level of the knee above the head of the fibula to the lateral malleolus. The skin and subcutaneous cellular tissues were reflected, the muscular fascia was exposed, and the muscles were carefully retracted to expose the vascular-nervous pedicle (Figs. 2 and 3).

### Measurements of the peroneal longus and peroneal brevis muscles and delimitations of the quadrants

**Figure 4:**
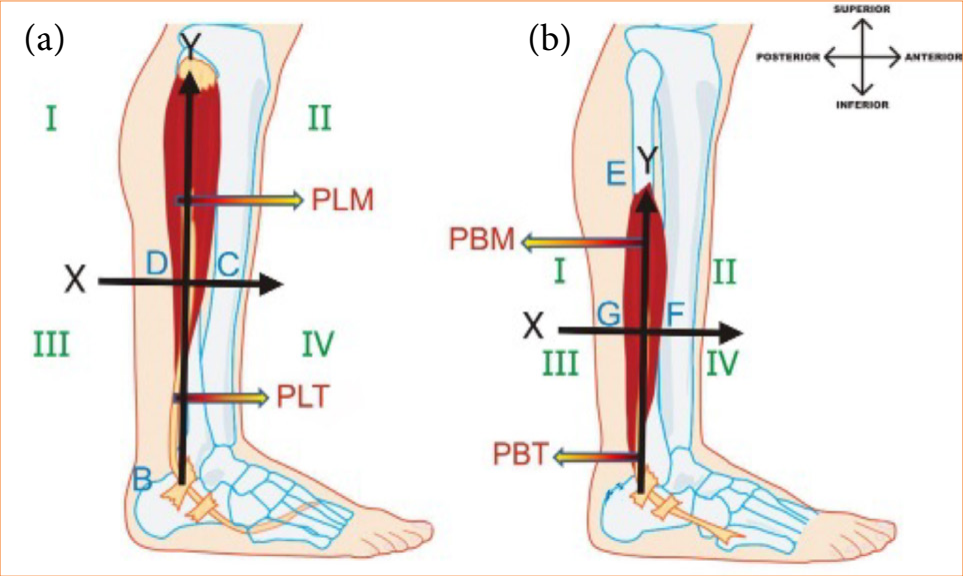
Diagram illustrating the quadrants idealized in this work: left PLM and left PBM. (a) Lateral condyle; (b) lateral malleolus. PLM: a longitudinal line through the lateral condyle of the fibula (A) to point B on the lateral malleolus, called AB (axis y), and a second line perpendicular to the first, crossing the midpoint of the transverse line (CD, axis x); PBM: the y-axis is formed by drawing a line from point E, which originates from the distal two-thirds of the lateral surface of the fibula, to point B at the lateral malleolus, creating segment EB. A transverse line is also drawn, which is perpendicular to the y-axis, crossing the midpoint of the transverse line, forming segment FG, and giving rise to the x-axis.

PLM: peroneus longus muscle; PLT: peroneus longus tendon; PBM: peroneus brevis muscle; PBT: peroneus brevis tendon. Source: Elaborated by the author Nazareth Cristina Delantonia.

Morphometric measurements of the muscle dimensions (transverse and longitudinal) were performed (Fig. 4). We divided the entire area of the PL and PB muscles into four areas to describe the branching pattern of the SPN in the muscle: two superior (1, 2) and two inferior (3, 4) areas according to the two axes (x, y) (Fig. 4). These four areas were based on two reference lines: the PL muscle, a longitudinal line through the lateral condyle of the fibula (A) to point B on the lateral malleolus, called AB (axis Y), and a second line perpendicular to the first, crossing the midpoint of the transverse line (CD, axis x) (Fig. 4). The y-axis was formed by drawing a line from point E, which originates from the distal two-thirds of the lateral surface of the fibula, to point B at the lateral malleolus, creating segment EB. A transverse line perpendicular to the y-axis was also drawn, crossing the midpoint of the transverse line forming segment FG and giving rise to the x-axis.

By convention, the following were adopted: the intersection of the axes as the origin and zero point; the upper posterior quadrant with negative abscissa and positive ordinate, called quadrant I; the upper anterior quadrant with positive abscissa and positive ordinate, called quadrant II; the lower posterior quadrant with negative abscissa and negative ordinate, called quadrant III; and the lower anterior quadrant with positive abscissa and negative ordinate, called quadrant IV (Fig. 4).

The data were grouped into categories, forming four areas of distribution to facilitate clinical correlation. The middle transverse line separates the upper and lower areas and is divided into four equally sized segments. Perpendicular lines were drawn on these four segments, as shown in Fig. 4.

**Figure 5:**
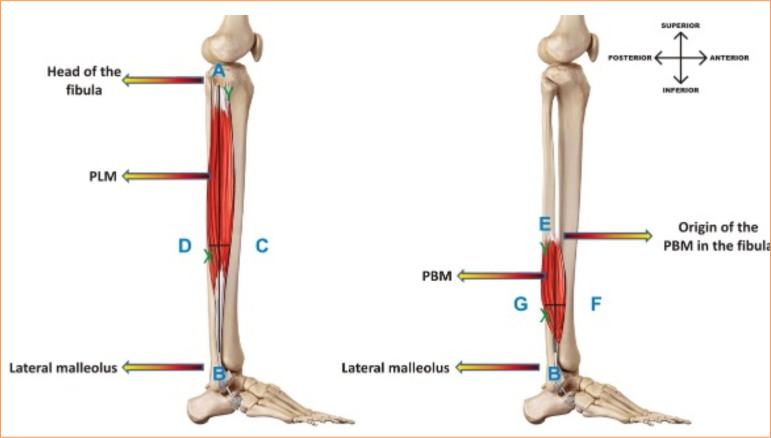
Peroneal muscles were measured according to the muscle transverse DC in peroneus longus muscle (PLM) and GF in peroneus brevis muscle (PBM), and longitudinal diameters AB in PLM and EB in PBM to delimit a plane.

Source: Elaborated by the author Nazareth Cristina Delantonia.

From the dimensions of the PL and PB muscles, the penetration points of the branches of the SPN in the PL and PB muscles were measured according to the transverse and longitudinal muscle diameters to delimit a plane. This method was previously described by Akamatsu et al.^23^ to evaluate the location of the penetration points of nerve branches in the muscle belly using anatomical dissection2^3^ (Fig. 5).

**Figure 6:**
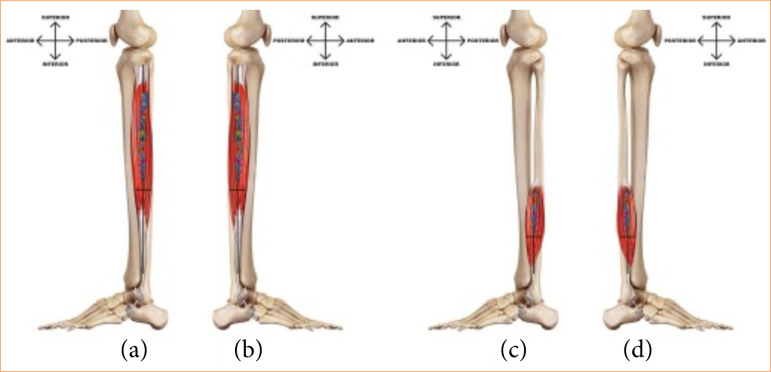
Diagram illustrating the measured points and idealized quadrants in this work. (a) Left PLM; (b) right PLM; (c) left PBM; (d) right PBM.

PLM: peroneus longus muscle; PBM: peroneus brevis muscle. Source: Elaborated by the author Nazareth Cristina Delantonia.

**Figure 7:**
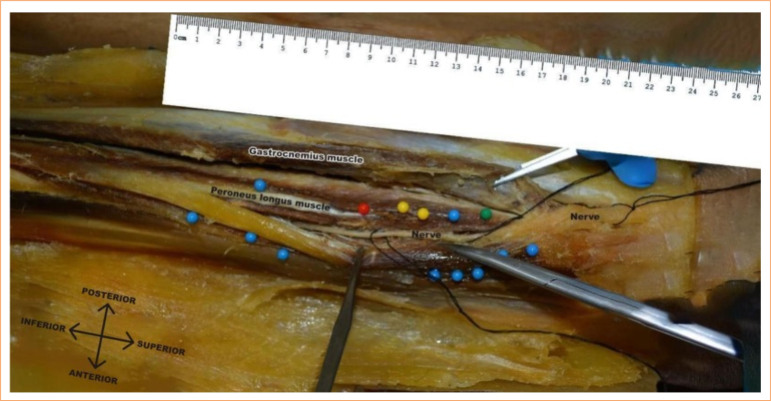
Illustration of the marking of the entry points of the superficial peroneal nerve into the peroneus longus muscle (right leg, decubitus ventral).

Source: Elaborated by the author Flavia Emi Akamatsu Jacomo.

The points of entry of the SPN branches into the muscles were indicated with pins (Figs. 6 and 7) and documented photographically using a Nikon D52 camera (Nikon Corporation, Tokyo, Japan). The penetration points were measured in relation to the median longitudinal and transverse axes by a simple division of values and classified according to the numbered areas from I to IV.

### Statistical analysis

#### Sample calculation

The sample calculation was based on the results of the first five cadavers (10 muscles) evaluated in the pilot project. The difference obtained between quadrants I and IV for the PB muscle was an average of 2.50 points with a variability of 2 points (standard deviation [SD] = 2 points). To detect the difference observed in the pilot sample with 80% power and 95% confidence, the sample required for conducting the study was 15 muscles, considering a two-tailed test^24^. The sample calculation was based on the difference between quadrants of the PB muscle, as the difference observed in the pilot sample was smaller than the difference observed in the PL muscle, which was 5.6 points between quadrants I and III, with a variability of 2.2 points.

Because our findings demonstrated greater variation than one entry point, our sample reflected the expected results from a larger population. This was considered in the two-sided test calculation. For sex, muscles were described using summary measures, and comparisons were made using Student’s t-tests^25^. Mann–Whitney’s tests were used to compare the total number of points in each individual muscle according to sex, and the paired Wilcoxon’s test^25^ was used to compare the number of points between sides. The number of points related to each area analyzed was described and compared between areas using generalized estimation equations with an interchangeable correlation matrix among sides and sextants, using a Poisson marginal distribution and identity link function^26^, followed by multiple Bonferroni’s comparisons^24^ to identify areas (I–IV) that showed differences. Pearson’s and Spearman’s correlation methods^27^ were employed to assess the association between anthropometric descriptors and the number of SPN entry points in the PL and PB muscle bellies. Analyses were performed using IBM Statistical Package for the Social Sciences Statistics for Windows, version 26.0 (IBM Corporation, Armonk, NY, United States of America). The tests were performed at a significance level of 5% (*p* < 0.05).

## Results

The ages of the cadavers were 66–92 years old (mean = 78.1). The approximate heights ranged 1.60–1.77 m (mean = 1.68).

The weight and body mass index were 46–85 kg (mean = 65.95) and 15.9–33.2 kg/m^2^ (mean = 21.33), respectively. Eight of the nine cadavers were white, and the remaining one was non-white (Table 1).

**Table 1:** Description of the characteristics of the cadavers<tfn>*</tfn>.

Variable	Description (N = 12)
**Age (years old)**	
Average ± SD	78.4 ± 12.8
Median (p25; p75)	79 (66; 91)
**Sex**	
Feminine	8 (66.7)
Masculine	4 (33.3)
**BMI (kg/m** ^2^ **)**	**(N = 8)**
Average ± SD	21.6 ± 5.8
Median (p25; p75)	21.7 (16.5; 24.1)

* Data were not obtained from any of the cadavers as four were incomplete. Therefore, the sample size was eight participants; SD: standard deviation; BMI: body mass index. Source: Division of Human Structural Anatomy, Department of Surgery.

Measurements of longitudinal (AB) and transverse (CD) muscle dimensions according to sex revealed no significant differences (*p* > 0.05) between PLM and PBM. No significant differences were observed between the other characteristics in terms of sex or side (*p* > 0.05) for PL and PB muscles (Tables 2 and 3).

**Table 2:** Description of muscle measurements according to sex and results of comparisons<tfn href="tfn01">*</tfn>.

Variable	Sex		*p* -value
	Feminine	Masculine	
**Right PL LA-LB (cm)**			**0.040**
Average ± SD	32.3 ± 2.3	35.8 ± 2.3	
Median (p25; p75)	33 (30; 34)	35.8 (33.6; 37.9)	
**Left PL LA-LB (cm)**			0.148
Average ± SD	33.4 ± 2.8	36.1 ± 3.1	
Median (p25; p75)	33 (30.5; 36)	36.3 (33.3; 38.9)	
**Right PL LC-LD (cm)**			0.063
Average ± SD	2.8 ± 1	1.9 ± 0.2	
Median (p25; p75)	3 (2; 4)	2 (1.8; 2)	
**Left PL LC-LD (cm)**			0.885
Average ± SD	2.6 ± 0.7	2.5 ± 0.6	
Median (p25; p75)	2.3 (2; 3)	2.5 (2; 3)	
**Right PB BE-BB (cm)**			0.266
Average ± SD	21.3 ± 2.3	23.1 ± 2.8	
Median (p25; p75)	21 (19; 24)	24 (20.3; 25.1)	
**left PB BE-BB (cm)**			0.907
Average ± SD	20.3 ± 3	20 ± 4.2	
Median (p25; p75)	20 (17.8; 23.5)	21.5 (15.5; 23)	
**Right PB BF-BG (cm)**			0.733
Average ± SD	2.2 ± 0.8	2.4 ± 0.7	
Median (p25; p75)	2.5 (1.5; 3)	2.5 (1.7; 3)	
**Left PB BF-BG (cm)**			0.772
Average ± SD	2.2 ± 0.6	2.3 ± 0.5	
Median (p25; p75)	2.1 (2; 2.9)	2.3 (1.9; 2.9)	
**Right PL points**			0.230£
Average ± SD	9.6 ± 2.4	11 ± 1.6	
Median (p25; p75)	10 (8; 10)	11 (9.5; 12.5)	
**Left PL points**			0.368£
Average ± SD	9.1 ± 2.1	12 ± 5.4	
Median (p25; p75)	9 (8; 10.5)	10 (8.5; 17.5)	
**Right PB points**			0.230£
Average ± SD	5.4 ± 1.1	4.5 ± 0.6	
Median (p25; p75)	6 (4; 6)	4.5 (4; 5)	
**Left PB points**			0.570£
Average ± SD	5 ± 1.3	5.8 ± 2.2	
Median (p25; p75)	4.5 (4; 6.5)	5 (4.3; 8)	

* Paired Student’s t-test; £: unpaired Mann-Whitney’s U test; PL: peroneus longus; SD: standard deviation; PB: peroneus brevis. Source: Elaborated by the authors.

**Table 3:** Description of muscle measurements according to sides and the results of comparisons<tfn href="tfn01">*</tfn>.

Variable	Side		*p* -value
	Right	Left	
**PL LA-LB (cm)**			0.262
Average ± SD	33.5 ± 2.8	34.3 ± 3	
Median (p25; p75)	34 (31; 35)	34 (32; 36.8)	
**PL LC-LD (cm)**			0.551
Average ± SD	2.5 ± 0.9	2.5 ± 0.7	
Median (p25; p75)	2 (2; 3)	2.3 (2; 3)	
**PB BE-BB (cm)**			0.103
Average ± SD	22 ± 2.5	20.2 ± 3.2	
Median (p25; p75)	22.5 (19; 24)	20.5 (17.8; 23)	
**PB BF-BG (cm)**			> 0.999
Average ± SD	2.3 ± 0.7	2.3 ± 0.6	
Median (p25; p75)	2.5 (1.5; 3)	2.1 (2; 2.9)	
**PL points**			0.788**
Average ± SD	10.1 ± 2.2	10.1 ± 3.6	
Median (p25; p75)	10 (9; 11)	9 (8; 10.8)	
**PB points**			0.565**
Average ± SD	5.1 ± 1	5,3 ± 1.6	
Median (p25; p75)	5 (4; 6)	5 (4; 6.5)	

* Paired Student’s t-test;

** unpaired Wilcoxon’s test; PL: peroneus longus; PB: peroneus brevis; SD: standard deviation. Source: Elaborated by the authors.

**Figure 8:**
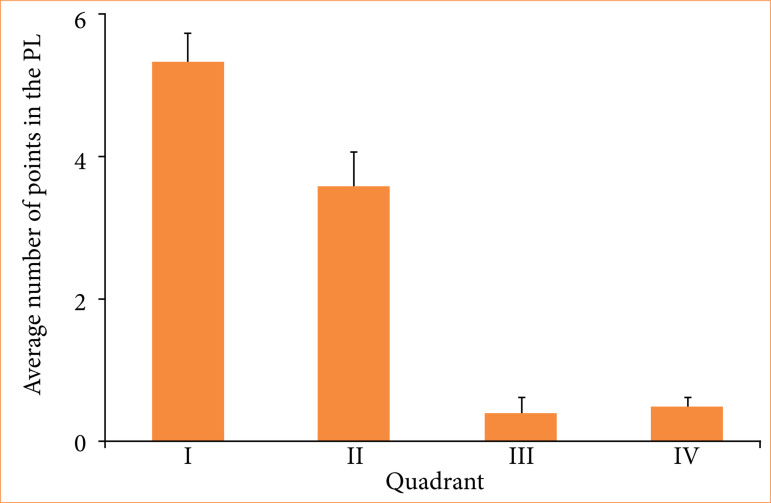
Average number of points in the PL according to quadrants with the respective standard errors.

**Figure 9:**
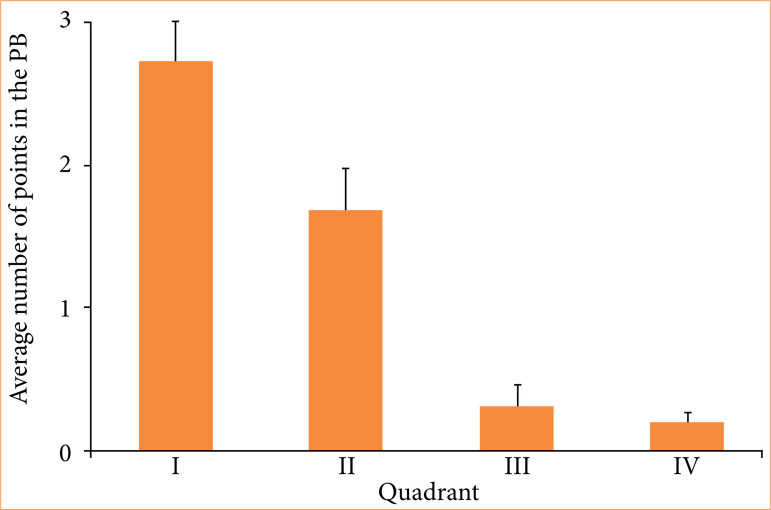
Average number of points in the PB according to quadrants with the respective standard errors.

It also observed that the transverse dimension of the PL muscle correlated with body mass index on the right side(r = 0.909; *p* = 0.005) (Table 4). The points of entry of the SPN into the muscle belly were observed in all quadrants of all cadavers, regardless of sex or side. However, the distribution of the SPN entry points among the quadrants varied significantly (*p* < 0.001) (Table 5; Figs. 8 and 9), with a higher concentration in the upper quadrant.

**Table 4:** Results of the correlations of the quantitative parameters of the cadavers with the measurements in the muscles and the total number of points<tfn href="tfn01">*</tfn>.

Correlation		Age (years old)	BMI (kg/m^2^)
Right PL LA-LB (cm)	r	-0.357	-0.504
	p	0.281	0.249
Left PL LA-LB (cm)	r	-0.276	-0.070
	p	0.386	0.869
Right PL LC-LD (cm)	r	0.356	0.909
	p	0.283	0.005
Left PL LC-LD (cm)	r	0.019	0.507
	p	0.952	0.200
Right PB BE-BB (cm)	r	0.039	-0.028
	p	0.909	0.952
Left PB BE-BB (cm)	r	0.113	0.323
	p	0.727	0.436
Right PB BF-BG (cm)	r	-0.246	0.284
	p	0.466	0.536
Left PB BF-BG (cm)	r	0.010	0.024
	p	0.975	0.955
Right PL points*	r	0.229	-0.468
	p	0.498	0.289
Left PL points*	r	-0.586	-0.469
	p	0.045	0.241
Right PB points*	r	0.529	-0.217
	p	0.094	0.641
Left PB points*	r	-0.084	-0.350
	p	0.796	0.395

* Pearson’s correlation;

**Spearman’s correlation; r: the correlation coefficient, which ranges from -1 (an inverse correlation) to 1 (a direct correlation); p: whether the correlation is statistically significant; PL: peroneus longus; PB: peroneus brevis; BMI: body mass index. Source: Elaborated by the authors.

**Table 5:** Description of the number of points in each muscle according to quadrants and the results of comparisons<tfn href="tfn01">*</tfn>.

Variable	Quadrant				*p* -value
	I	II	III	IV	
**PL points**					**< 0.001**
Average ± SD	6 ± 3	4 ± 3	1 ± 1	1 ± 1	
Median (p25; p75)	5 (4; 7)	4 (2; 6)	0 (0; 0)	0 (0; 1)	
**PB points**					**< 0.001**
Average ± SD	3 ± 2	2 ± 2	1 ± 1	0 ± 1	
Median (p25; p75)	3 (2; 5)	2 (0; 3)	0 (0; 0)	0 (0; 0)	

* EEG with Poisson distribution and identity link function, assuming an exchangeable correlation matrix between sides and quadrants; PL: peroneus longus; PB: peroneus brevis; SD: standard deviation.

Source: Elaborated by the authors.

PL: peroneus longus. Source: Elaborated by the authors.

PB: peroneus brevis. Source: Elaborated by the authors.

The number of points differed significantly between the quadrants of the PL and PB muscles (*p* < 0.001), with a greater number of points observed in quadrants I and II compared with quadrants III and IV (Table 5).

The average differences in the number of points did not differ significantly for quadrants I *versus* II and quadrants III *versus* IV (*p* > 0.05) (Table 6), while the greater number of points was significantly higher in quadrants I and II than in quadrants III and IV (*p* < 0.001) (Figs. 8 and 9).

**Table 6:** Comparisons between quadrants of the number of muscle points<tfn href="tfn01">*</tfn>.

Variable	Comparison of quadrants		Average difference	Standard error	*p* -value	95%CI	
						Lower	upper
PL points	I -	II	1.61	0.60	0.042	0.03	3.18
	I -	III	4.96	0.49	< 0.001	3.67	6.25
	I -	IV	4.91	0.49	< 0.001	3.62	6.21
	II -	III	3.35	0.41	< 0.001	2.26	4.44
	II -	IV	3.30	0.42	< 0.001	2.21	4.40
	III -	IV	-0.04	0.19	> 0.999	-0.54	0.45
PB points	I -	II	1.22	0.46	0.049	0.00	2.43
	I -	III	2.57	0.38	< 0.001	1.55	3.58
	I -	IV	2.70	0.38	< 0.001	1.71	3.68
	II -	III	1.35	0.31	< 0.001	0.54	2.15
	II -	IV	1.48	0.29	< 0.001	0.70	2.25
	III -	IV	0.13	0.16	> 0.999	-0.30	0.56

* Bonferroni’s multiple comparisons; PL: peroneus longus; PB: peroneus brevis; 95%CI: 95% confidence interval. Source: Elaborated by the authors.

## Discussion

This study related the entry points of the superficial fibular nerve to the locations of MTPs in the PL and PB muscles. The same reasoning was followed in previous studies conducted by our group on the innervation of the trapezius, gluteus maximus, abductor hallucis, masseter, and temporal muscles^23,28-31^. Previous studies have suggested some variations in the distribution areas, but the description of MTPs reported by Travell and Simons5 was considered in the present study because no precise previous data were found regarding their locations in PL and PB muscles.

The data obtained in this study through the observation and classification of SPN entry sites into four quadrants in dissected specimens showed variability in the number of SPN insertions (the difference ranged from one to six insertion points for PL muscle and one to three for PB muscle), with no difference between the right and left sides.

Therefore, it can be concluded that despite the large variability among the specimens, the number of SPN entry points in each specimen was independent of laterality.

The distribution of SPN entry points in the four quadrants was established in PL and PB muscles and did not show a statistically significant difference between male and female specimens. However, a statistically significant difference was observed in the number of superficial fibular nerve entry sites (*p* < 0.05).

We found that the insertion points of the SPN, predominantly in regions I and II, reflected the location of TP in the region of entry of the nerve into the muscle, as described by Duchenne de Boulogne^32^, Remak^33^, and Ziemssen^34^. In a study by Happak et al.^35^, motor plates were located near nerve entry points in the muscle. In our study, regions I and II were the locations of the SPN entering the PL and PB. Dividing the muscle into quadrants possibly allows for a more precise localization of the TPs.

We observed that in all PL and PB muscles, the nerve runs parallel to the muscle, sending its branches to the muscle belly like “tree branches.” In a work by Pinheiro et al.^30^, it was reported that the nerve crosses the masseter muscle longitudinally in a diagonal direction. In the gluteus maximus muscle^23^, there is a fan-like branching of the nerve that is distributed throughout the muscle. Therefore, we suggest that the pattern of innervation is related to the shape and function of the muscles. From the beginning of muscle development, some properties are determined, such as the anatomical structure of the muscle, including the shape, spatial organization, and orientation of fascicle components and connections with tendons, bones, and aponeuroses^36^. The shape of PL and PB muscles must be optimal to fulfill their function, mainly as ankle evertors^37^ and plantar flexors.

This study is useful for determining the precise locations of pain origins and for tailoring treatment approaches. The anatomical localization of myofascial points should be used to assist research using ultrasonography^20^ and MRI^21^ to assess the accuracy of diagnostic tests and the repeatability of data, thereby determining the most effective approaches. Finding quadrants I and II with more nerve entry sites on PL and PB muscles may help guide these examinations. Methods such as acupuncture, shockwave therapy, local anesthetic injections, dry needling, intramuscular electrical stimulation^38^, and various other therapies have been used to alleviate symptoms associated with abnormalities in MTPs, with some authors reporting therapeutic benefits^22,39-42^.

### Study limitations

We were unable to develop age- and race-based groups because the cadavers we had access to were from the anatomy acquisition and unavailable for selection. Since it is not possible to dissect a living person, this method cannot connect MTPs, which would provide a better understanding of the pathogenesis and diagnosis of myofascial disorders. Due to the limited number of donations in our country, the statistician determined a minimum sample size adequate to represent the general population in accordance with the study’s objective, which was to establish a link between MTPs and superficial fibular nerve entry points.

## Conclusion

The clinically described MTPs responsible for MPS in the lateral compartment region of the leg are anatomically related to the distribution pattern of SPN branches in PL and PB muscles. MTPs are described in the literature, but they were not directly assessed in the present study.

## Data Availability

The data will be available upon request.
